# Prevalence of Migraine in a Convenience Sample of Children Aged 5-12 Years in Jeddah, Saudi Arabia

**DOI:** 10.7759/cureus.117220

**Published:** 2026-09-30

**Authors:** Amr Morsy, Muhsen Alamoudi, Amjad Bahaj, Yousef Alkhaldi, Abdulrhman Tubagy, Ahmed Altowairqi, Shereen M Elghany

**Affiliations:** 1 Pediatric Medicine, Ibn Sina National College for Medical Studies, Jeddah, SAU; 2 Medicine, Ibn Sina National College for Medical Studies, Jeddah, SAU; 3 Pediatrics, Ibn Sina National College for Medical Studies, Jeddah, SAU

**Keywords:** child, epidemiology, family history, headache, migraine disorders, pediatric migraine, prevalence, questionnaires, saudi arabia, school-aged children

## Abstract

Background: Migraine is a common neurological disorder in children and adolescents that may affect school attendance, daily functioning, and quality of life. Contemporary data regarding migraine prevalence among younger school-aged children in Jeddah, Saudi Arabia, are lacking. This study aimed to estimate the 12-month prevalence of questionnaire-defined migraine among children aged 5-12 years in Jeddah, Saudi Arabia.

Methods: A cross-sectional study was conducted among community-recruited children aged 5-12 years residing in Jeddah, Saudi Arabia, between March 2026 and July 2026. Participants were recruited through convenience sampling using online parent and school-related social media groups. Parents or legal guardians were asked to complete the McMaster Pediatric Migraine Questionnaire. Participants who reported headache during the preceding 12 months were classified according to the questionnaire's predefined scoring algorithm based on the International Classification of Headache Disorders, Second Edition (ICHD-II) criteria. The 12-month prevalence of questionnaire-defined migraine was calculated as the proportion of participants fulfilling the predefined questionnaire criteria, with an exact 95% confidence interval. Associations with categorical variables were assessed using Pearson's chi-square or Fisher's exact test.

Results: Among 183 children, 130 (71.0%) were male, and 53 (29.0%) were female. Sixty-nine participants (37.7%) reported headache during the preceding 12 months, and 27 children (14.8%; 95% CI: 10.0-20.7%) met the questionnaire criteria for migraine. Migraine status was not significantly associated with sex or age (p = 0.261 and p = 0.226, respectively). Among children with headache, family history of migraine was significantly associated with migraine status (p < 0.001); those reporting a positive family history had higher odds of questionnaire-defined migraine (OR = 7.92, 95% CI: 2.49-25.22).

Conclusion: In this study of children aged 5-12 years in Jeddah, 27 of 183 participants met the predefined questionnaire criteria for migraine, corresponding to a 12-month questionnaire-defined migraine prevalence of 14.8% (95% CI: 10.0-20.7%). Findings should be interpreted cautiously given the convenience sampling, reliance on parental questionnaire responses, the long recall period, and the use of an Arabic-translated, not formally validated questionnaire based on ICHD-II criteria rather than contemporary clinical assessment. Population-based studies using validated Arabic instruments and current diagnostic criteria are warranted.

## Introduction

Migraine is considered a leading neurological disorder affecting children and adolescents [1,2]. Patients with migraine suffer recurrent episodes of moderate-to-severe head pain often accompanied by nausea, photophobia, and phonophobia [3-5].

Migraines can negatively impact school performance and quality of life [6]. Pediatric migraine is also associated with anxiety and sleep disturbance [7].

According to a recent systematic review and meta-analysis of individuals aged 8-18 years, it was found that the approximate pooled prevalence of migraine is about 11% [8]. Moreover, there was a 24.17% rise in global migraine cases among children and adolescents from 1990 to 2021 [1]. This high prevalence is observed globally, with the Middle East region being significantly affected [2].

Despite this substantial prevalence of migraine, it is still underdiagnosed [9]. The current study focuses on the age group of 5-12 years, as this period represents an important stage in childhood development and during which migraine may occur but can be difficult to diagnose [10,11]. Underdiagnosis at this age may be attributed to the difficulty of describing migraine symptoms at this particular age group [10] or due to lack of parental awareness [12,13].

In Jeddah, most epidemiological research focused on general or adult populations rather than school-aged children. A large cross-sectional study of 2,058 participants in Jeddah aged 18-60 years reported a migraine prevalence of 37.2%, with females and students exhibiting a higher rate of migraine [14].

Another cross-sectional study among health sciences college students in Jeddah documented an even higher prevalence (71.6%) among college-aged individuals [3].

Another study conducted in Riyadh reported an age-adjusted migraine prevalence of ~6.2% among children aged 6-15 years [15]. However, this study was conducted more than 20 years ago and in a different Saudi region, leaving limited contemporary evidence regarding migraine prevalence among younger school-aged children in Jeddah.

A recent Saudi systematic review highlights migraine as a major burden but does not provide prevalence estimates specifically for children aged 5-12 years in Jeddah [16].

Although migraine prevalence has been investigated among adults and university students in Jeddah and among school children in Riyadh, contemporary population-based data on migraine among children aged 5-12 years in Jeddah remain limited.

To our knowledge, published data specifically addressing migraine prevalence among children aged 5-12 years in Jeddah remain limited. Furthermore, diagnostic tools and methods vary widely across studies, hindering direct comparison of prevalence estimates across studies [8].

Given the limited contemporary data on migraine among children in Jeddah, Saudi Arabia, this study aimed to estimate the 12-month prevalence of questionnaire-defined migraine among children aged 5-12 years in Jeddah.

## Materials and methods

Study design

This study adopted a cross-sectional descriptive design to assess the prevalence of migraine among children aged 5-12 years in Jeddah, Saudi Arabia.

Study setting

The study was conducted in Jeddah, Saudi Arabia, over the period from March 2026 to July 2026. Data were collected from parents or guardians of children aged 5-12 years living in Jeddah. Participants were recruited using a convenience sampling approach through social media platforms, such as WhatsApp, Twitter/X, Telegram, and other parent or school-related online groups that were accessible to the research team.

Relevant online groups accessible to the research team and whose membership included parents, guardians, or families in Jeddah were identified, and then the invitation and survey link were shared on these groups.

The survey began with a question about the age of the child and the address to confirm participant eligibility. Responses from individuals who did not meet these criteria were excluded. To minimize duplicate responses, the online survey platform was configured, where technically possible, to limit multiple submissions from the same device, and responses were reviewed for potential duplicates before analysis.

Study population and sample size

The target population included children aged 5-12 years living in Jeddah. Data were obtained from their parents or legal guardians, who served as the study respondents. The minimum required sample size was estimated using the following formula [17]:

*n = (Z&sup2; &times; p &times; (1&minus;p)) / d&sup2;*

Here, n = required sample size, Z = Z value for confidence level 95% confidence (1.96), p = expected prevalence = 0.062 [15], and d = desired precision (margin of error) = 0.05, so n = 90.

The expected prevalence was set at 6.2%, based on a study done in Riyadh to estimate migraine prevalence among school children [15], as this represents a comparable pediatric population in Saudi Arabia.

The minimum calculated sample size was 90 participants. Assuming a 10% non-response or incomplete-response rate, the final calculated sample size was 100 participants. A total of 183 eligible participants were finally included in the study.

Additionally, 312 participants completed the questionnaire, but 129 participants were excluded as they were outside the target age group. Therefore, 183 eligible participants were included in the final analysis, exceeding the minimum required sample size.

Inclusion and exclusion criteria

The study included children aged 5-12 years who were living in Jeddah, Saudi Arabia. Children with a previously diagnosed secondary headache disorder were excluded from the study.

Data collection tool

Data were collected using the McMaster Pediatric Migraine Questionnaire (McMaster Migraine Tool), a screening instrument developed to assess migraine in children aged 5-12 years. The original instrument was developed based on the International Classification of Headache Disorders, Second Edition (ICHD-II) [18] criteria and was evaluated against assessment by a pediatric neurologist. In the original validation study, the McMaster Pediatric Migraine Questionnaire demonstrated a sensitivity of 84% and a specificity of 69% [10]. The questionnaire assessed sociodemographic characteristics, headache characteristics, family history of migraine, the functional impact of headache on daily activities, and headache red flags.

As the McMaster Pediatric Migraine Questionnaire was developed and validated using the ICHD-II criteria [18], questionnaire-defined migraine in the present study was interpreted as a screening outcome and was not considered equivalent to a diagnosis based on the current ICHD-III criteria [19].

Permission to use the McMaster Pediatric Migraine Questionnaire was obtained from the original author. To facilitate comprehension and completion by Arabic-speaking participants, the questionnaire was translated from English into Arabic using a forward-backward translation procedure. The translated version was subsequently piloted among 15 parents of children within the target age range to assess its clarity and comprehensibility. The pilot participants reported that the questionnaire items were generally clear and understandable, and no major comprehension problems were identified. Written confirmation was then obtained from the original author permitting the use of the Arabic-translated version for the present academic study.

No formal psychometric or diagnostic validation of the Arabic translation was conducted as part of the present study, and no formal cross-cultural adaptation study was undertaken beyond the translation, back-translation, and pilot assessment described above. Therefore, the pilot testing should be interpreted as an assessment of clarity and comprehensibility rather than validation of the Arabic version.

The performance characteristics are related to the original English questionnaire and cannot be assumed to apply to the Arabic translation used in the present study. Accordingly, the questionnaire-defined migraine classification in this study should be interpreted as a screening outcome rather than as a formally validated diagnostic determination.

Assessment of migraine

Migraine status was determined according to the predefined diagnostic classification incorporated into the McMaster Pediatric Migraine Questionnaire, which was developed based on ICHD-II migraine criteria [18]. Participants were categorized according to the questionnaire's predefined criteria as having migraine or not having migraine.

All participants were initially assessed for the presence of headache during the last 12 months. Among the 183 participants, 69 reported having headaches, and each participant with headache was evaluated against the questionnaire criteria for migraine with aura and migraine without aura. Participants who fulfilled all required diagnostic criteria were classified as having migraine, while those who did not fulfill the required criteria were classified as not having migraine. Participants who met the diagnostic criteria of probable migraine, based on the scoring system of the used questionnaire, were not considered as migraine patients in the current study.

The questionnaire scoring was performed for each child individually rather than by using individual questionnaire items as independent indicators of migraine.

Questionnaire items were coded according to the original McMaster scoring algorithm. For descriptive presentation, selected responses were grouped into clinically relevant categories; these descriptive groupings were not used independently to establish migraine status.

Data collection procedure

After obtaining approval from relevant authorities, questionnaires were distributed to parents. Electronically informed consent was obtained from the parent or legal guardian before access to the questionnaire. Only completed responses from parents of children aged 5-12 years were included.

Data analysis

Data were entered and analyzed using Statistical Product and Service Solutions (SPSS; IBM SPSS Statistics for Windows, Armonk, NY). Categorical variables were summarized as frequencies and percentages. Migraine prevalence was calculated as the proportion of participants fulfilling the predefined questionnaire criteria, with exact 95% confidence intervals. Associations between categorical variables were assessed using Pearson's chi-square test or Fisher's exact test. Statistical significance was defined as a two-sided p-value <0.05.

Ethical considerations

Ethical approval was obtained from Ibn Sina National College Institutional Review Board (Protocol Identification Number 051MP/CR19022026) on March 2, 2026. Electronic informed consent was obtained from parents or guardians, and confidentiality of participants' data was strictly maintained. Participants were assured of the voluntary nature of participation and their ability to withdraw from participation at any time with no consequences.

## Results

A total of 183 children aged 5-12 years were enrolled in the study. Of these 183, 130 (71.0%) were males, and 53 (29.0%) were females. Eighty-three participants (45.4%) were aged 5 to <8 years, while 100 (54.6%) were aged 8-12 years (Table 1).

**Table 1:** Demographic characteristics of the study participants.

Parameters	Gender		Total, n (%)
Age in Years	Males, n (%)	Females, n (%)	
5-less than 8	58 (31.7)	25 (13.7)	83 (45.4)
8-12 years	72 (39.3)	28 (15.3)	100 (54.6)
Total	130 (71)	53 (29)	183 (100)

Table 2 illustrates the responses of the 69 participants whose children experienced headache to the different questionnaire questions.

**Table 2:** Responses to the McMaster Questionnaire (headache characteristics).

Variable/Question	Categories	Number of children with headache (n=69)	%
What is the total number of headache attacks the child experiences?	5 times or less	53	76.8
	More than 5 times	16	23.2
On average, how long does the child's headache last if untreated or if treatment is unsuccessful?	30 minutes to 1 hour	35	50.7
	More than 1 hour	34	49.3
Which description best fits the child's headache pain?	Consistent with migraine	22	31.9
	Inconsistent with migraine	47	68.1
During an average headache, how does the child rate the pain severity from 1 to 10?	1-4	42	60.9
	5-10	27	39.1
Where does the child's headache usually occur?	Inconsistent with migraine	8	11.6
	Consistent with migraine	61	88.4
Does the child's headache get worse with physical activity?	Yes	40	58.0
	No	29	42.
When the child has a headache, does the child experience any of the following symptoms?	Photophobia or phonophobia	47	68.1
	Nausea or vomiting	15	21.8
	None and others	7	10.1
When the child has a headache, does the child experience aura?	Yes	48	69.6
	No	21	30.4

Of these 69 participants whose children experienced headache, 53 (76.8%) were males, and 16 (23.2%) were females. While 27 participants (39.1%) were aged five to less than eight years, 42 (60.9%) were aged eight years or more (Table 3).

**Table 3:** Frequency and percentage distribution of study variables for participants suffering headache.

Parameters	Gender		Total, n (%)
Age in Years	Males, n (%)	Females, n (%)	
5-less than 8	20 (29)	7 (10.1)	27 (39.1)
8-12 years	33 (47.8)	9 (13.1)	42 (60.9)
Total	53 (76.8)	16 (23.2)	69 (100)

Of the 27 participants whose children met the criteria for migraine based on the questionnaire used, 19 (70.4%) were males, and 8 (29.6%) were females. While nine participants (33.3%) were aged five to less than eight years, 18 (66.7%) were aged eight years or more (Table 4).

**Table 4:** Frequency and percentage distribution of study variables for participants suffering migraine.

Parameters	Gender		Total, n (%)
Age in Years	Males, n (%)	Females, n (%)	
5-less than 8	7 (25.9)	2 (7.4)	9 (33.3)
8-12 years	12 (44.5)	6 (22.2)	18 (66.7)
Total	19 (70.4)	8 (29.6)	27 (100)

There was no statistically significant association between sex and overall headache diagnosis category (migraine, non-migrainous headache, or no headache), χ² (2) = 2.69, p = 0.261; Cramér's V = 0.121. In the overall study population, questionnaire-defined migraine was identified in 19 of 130 males (14.6%, 95% CI: 9.6%-21.7%) and 8 of 53 females (15.1%, 95% CI: 7.9%-27.1%). Thus, the proportion of participants meeting the questionnaire criteria for migraine was similar in males and females (14.6% vs. 15.1%, respectively) (Table 5).

**Table 5:** Association between gender and headache diagnosis category. χ²(2) = 2.69, p = 0.261; Cramér's V = 0.121

Parameters	Gender		Total
Questionnaire-defined migraine diagnosis	Males, n (%)	Females (%)	
Migraine	19 (14.6)	8 (15.1)	27 (14.8)
Non-migrainous headache	34 (26.2)	8 (15.1)	42 (22.9)
No headache	77 (59.2)	37 (69.8)	114 (62.3)
Total	130 (100)	53 (100)	183 (100)

Among children who reported headache, however, questionnaire-defined migraine was present in 19 of 53 males (35.8%, 95% CI: 24.3%-49.3%) and 8 of 16 females (50.0%, 95% CI: 28.0%-72.0%). The relatively small number of female participants with headache limits the precision of this estimate and precludes meaningful interpretation of this difference as evidence of a sex-related association.

There was no statistically significant association between age group and the questionnaire-defined migraine diagnosis category (migraine, non-migrainous headache, or no headache), χ²(2) = 2.97, p = 0.226; Cramér's V = 0.127. Migraine was identified in 9 out of 83 children aged 5 to <8 years (10.8%) and 18 out of 100 children aged 8-12 years (18.0%) (Table 6).

**Table 6:** Association between age and headache diagnosis category. χ²(2) = 2.97, p = 0.226; Cramér's V = 0.127

Parameters	Age in years		Total
Questionnaire-defined migraine diagnosis	5-less than 8, n (%)	8-12 years, n (%)	
Migraine	9 (10.8)	18 (18)	27 (14.8)
Non-migrainous headache	17 (20.5)	25 (25)	42 (22.9)
No headache	57 (68.7)	57 (57)	114 (62.3)
Total	83 (100)	100 (100)	183 (100)

Among the 69 children who reported headaches, 37 (53.6%) reported a positive family history of migraine, and 32 (46.4%) did not report a family history of migraine. A statistically significant association was observed between family history of migraine and migraine status among children with headache (χ² (1) = 12.06, p < 0.001). Children with a positive family history had higher odds of meeting the questionnaire criteria for migraine than children without a family history (OR = 7.92, 95% CI: 2.49-25.22) (Table 7).

**Table 7:** Association between family history of migraine and migraine diagnosis among children with headache. χ²(1) = 12.06, p < 0.001; OR = 7.92 (95% CI: 2.49-25.22)

Parameters	Family history of migraine		Total
Migraine status among children with headache	Positive, n (%)	Negative, n (%)	
Migraine	22 (59.5)	5 (15.6)	27 (39.1)
Non-migrainous headache	15 (40.5)	27 (84.4)	42 (60.9)
Total	37 (100)	32 (100)	69 (100)

Table 8 summarizes the chi-square, Cramér's V, and odds ratio statistics for the three associations examined above (sex, age group, and family history of migraine).

**Table 8:** Summary of statistical tests for associations with migraine diagnosis.

Association tested	χ²	df	p-value	Cramér's V	OR (95% CI)
Gender versus diagnosis category (migraine/non-migrainous headache/no headache)	2.69	2	0.261	0.121	-
Age group versus diagnosis category (migraine/non-migrainous headache/no headache)	2.97	2	0.226	0.127	-
Family history versus migraine status (among children with headache)	12.06	1	<0.001	-	7.92 (2.49-25.22)

Figure 1 illustrates the whole selection and results processing of the study.

**Figure 1:**
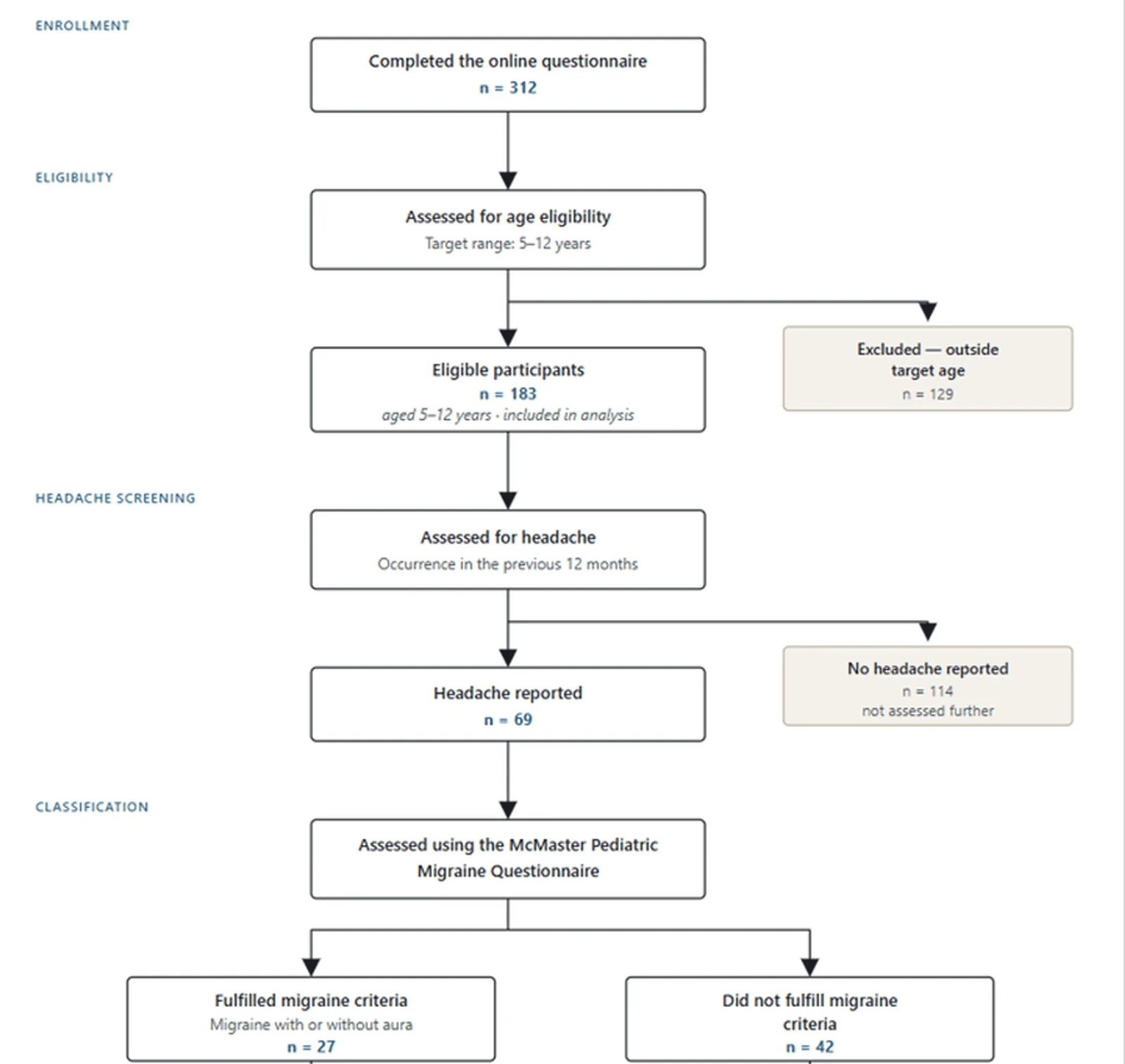
Summary of participants selection and results.

Among the 183 participants, 69 (37.7%) (95% CI: 30.7-44.7%) reported experiencing headache during the preceding 12 months. These participants were subsequently assessed using the predefined scoring algorithm of the McMaster Pediatric Migraine Questionnaire.

Overall, 27 (14.8%) out of the 183 participants met the questionnaire criteria for migraine, corresponding to a prevalence of 14.8% (95% CI: 10.0%-20.7%) (Figure 2). Of the 27 children meeting the questionnaire criteria for migraine, 25 (13.7% of the total study population) fulfilled the predefined criteria for migraine with aura, and 2 (1.1% of the total study population) fulfilled the criteria for migraine without aura (Figure 2).

**Figure 2:**
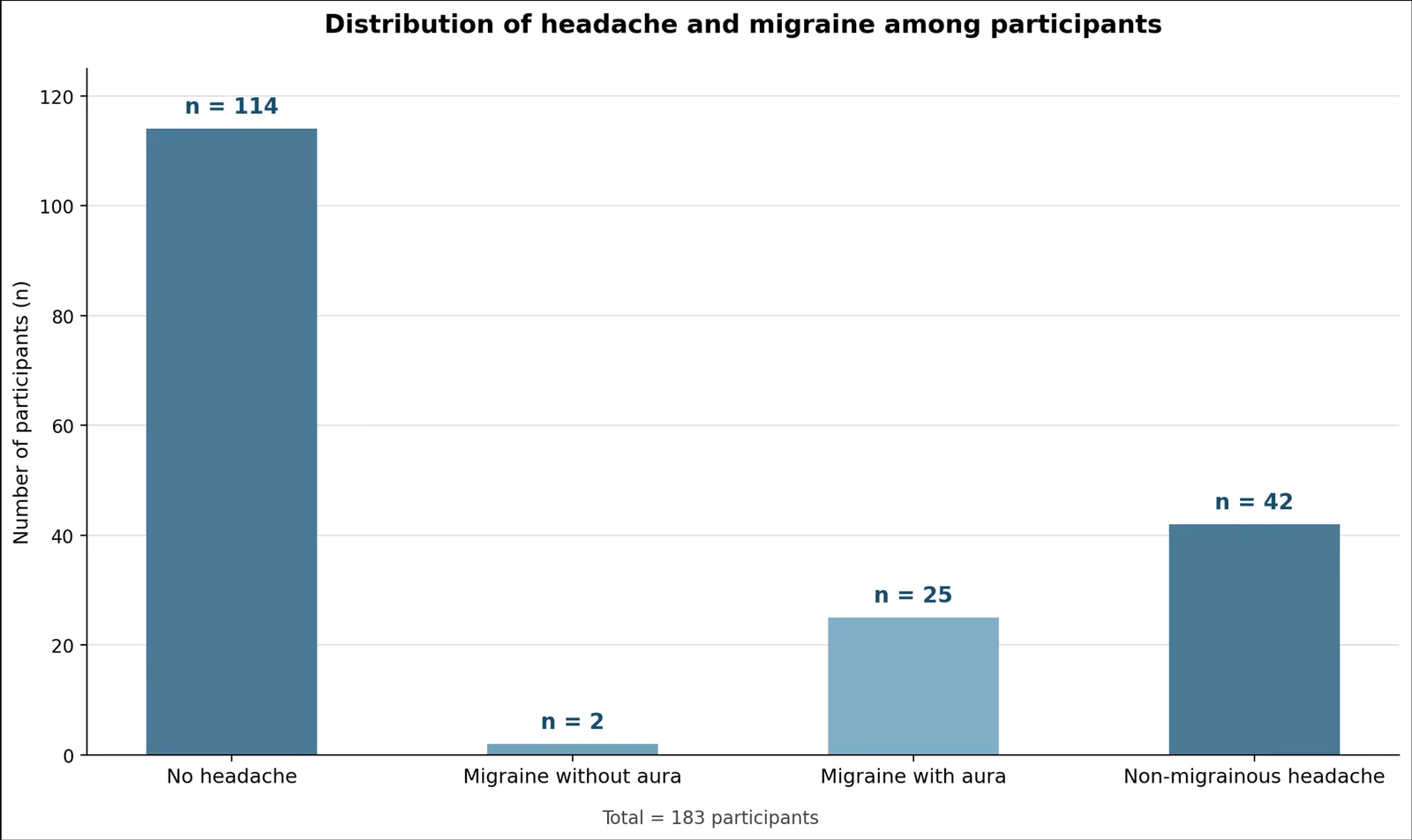
Distribution of headache and migraine among participants.

Among the 69 children who reported headache, 27 (39.1%) met the questionnaire criteria for migraine (Figure 3).

**Figure 3:**
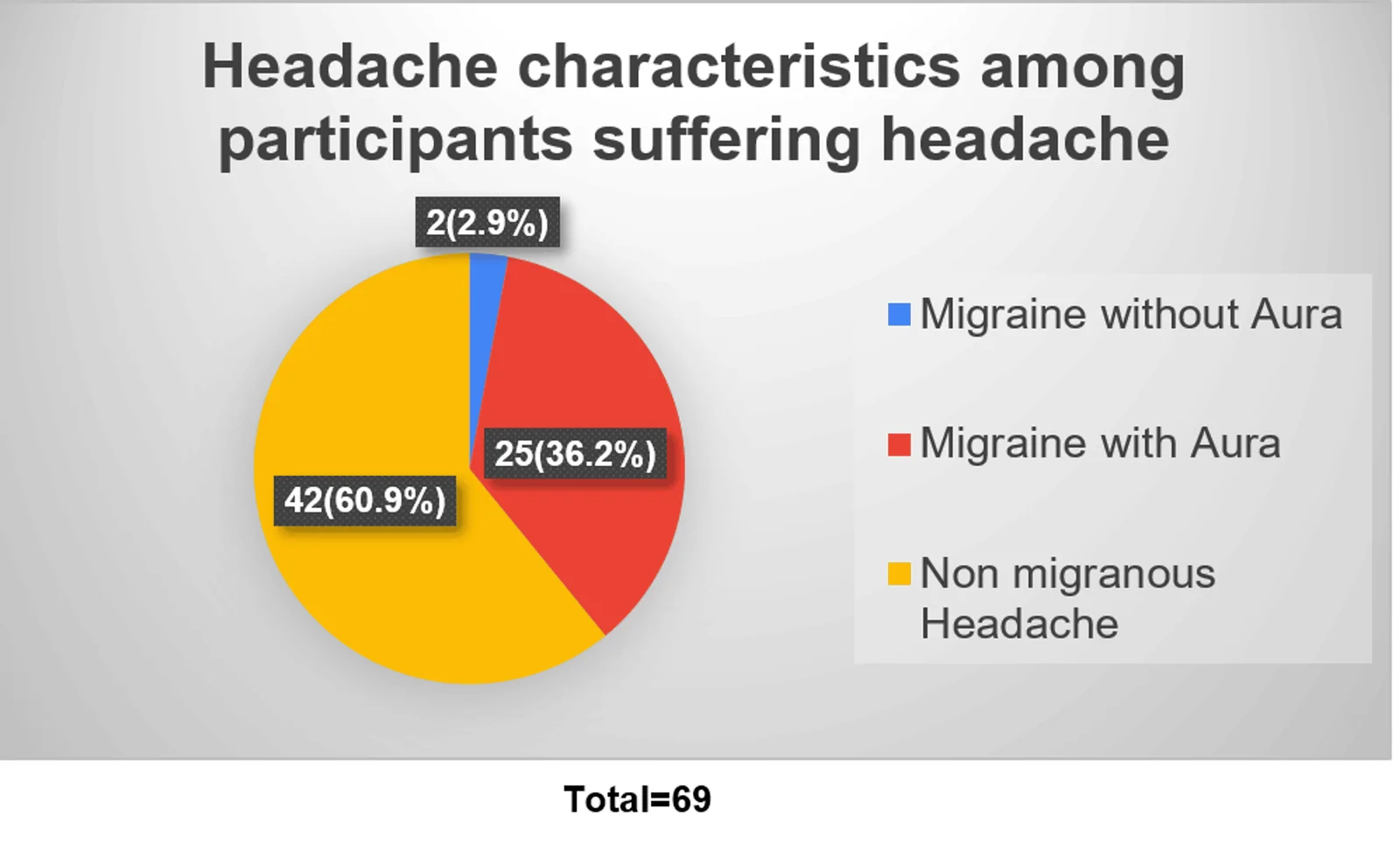
Headache characteristics among participants suffering headache.

Among the 27 participants who met the diagnostic criteria for migraine, 18 (67%) reported that migraine sometimes affects their daily activity, and 7 (26%) reported that their daily activity is often compromised by migraine. Only two participants (7%) reported that migraine never affects their daily activity (Figure 4).

**Figure 4:**
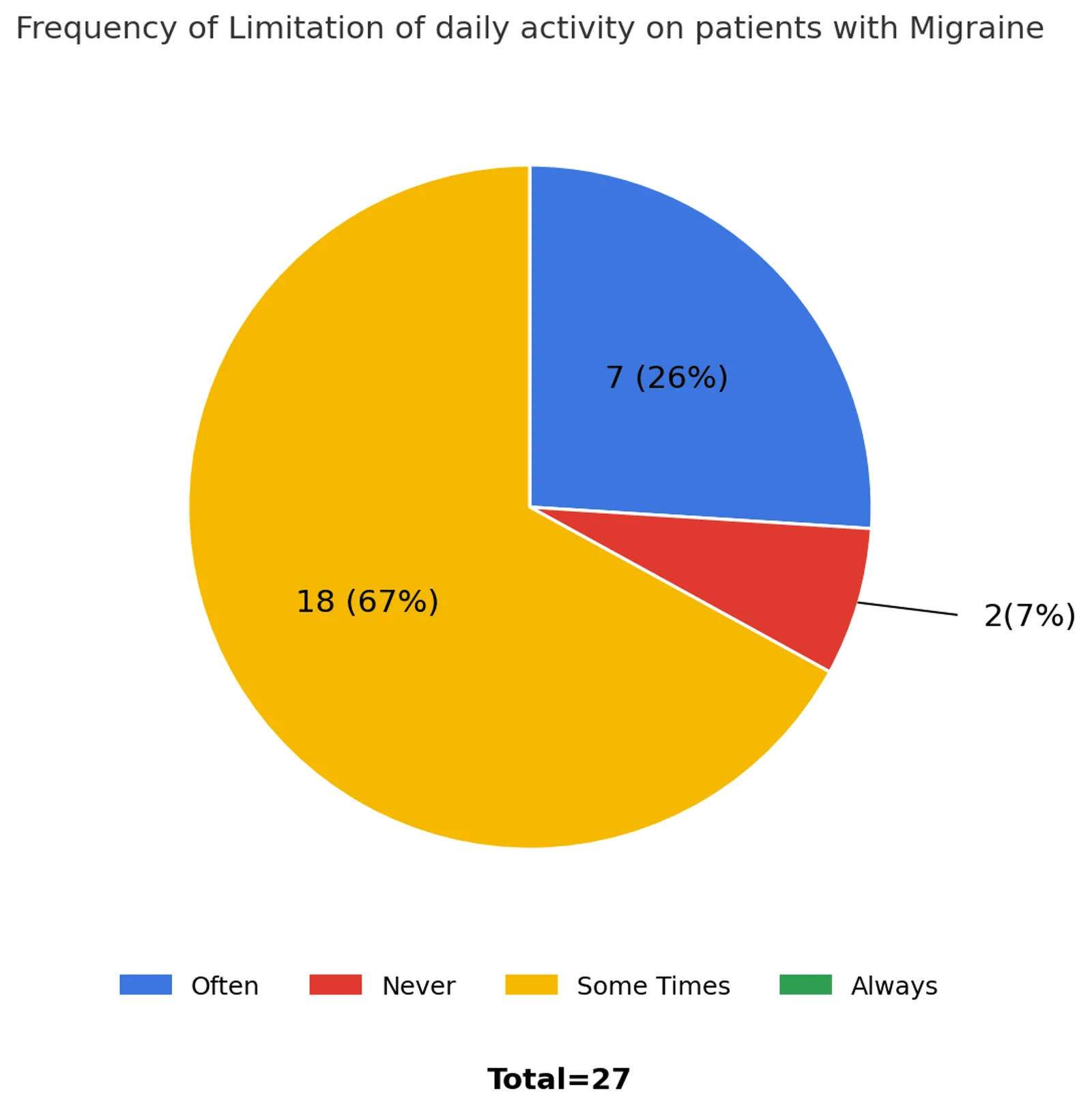
Impact of migraine on daily activities.

## Discussion

The present study found that 69 of 183 children aged 5-12 years (37.7%) were reported to have experienced headache during the preceding 12 months, while 27 children (14.8%) met the predefined questionnaire criteria for migraine. Among children who reported headache, 27 (39.1%) met the questionnaire criteria for migraine. These findings indicate that headache symptoms were relatively frequent in this sample of children in Jeddah, with a substantial proportion of those reporting headache meeting the questionnaire-based criteria for migraine. However, the findings should be interpreted in the context of the study design, sampling method, and diagnostic approach.

The observed proportion of children meeting the questionnaire criteria for migraine (14.8%) should be interpreted cautiously and should not be considered a population prevalence estimate for children in Jeddah. The use of convenience sampling and online recruitment may have resulted in a sample that differs systematically from the wider pediatric population. In addition, differences in age range, sampling methods, and diagnostic instruments make direct comparisons with studies conducted among adults or university students difficult. For example, previous studies from Jeddah have reported substantially higher migraine estimates among college students (71.6%) and adults aged 18-60 years (37.2%) [3,14]. These estimates are not directly comparable with the present findings because of the markedly different age groups and methodological approaches.

Greater relevance can be obtained from pediatric studies. In Riyadh, an age-adjusted migraine prevalence of 6.2% was reported among children aged 6-15 years [15]. Although this population is more comparable in age to the present study, differences in diagnostic instruments and study methodology still limit direct quantitative comparison. Taken together, the available Saudi studies suggest that estimates of migraine frequency vary considerably across populations, emphasizing the importance of age-appropriate, standardized diagnostic methods and representative sampling when comparing prevalence estimates.

An important finding was the relatively high frequency of reported headache during the preceding 12 months, with more than one-third of the participating children reporting headache. This finding may reflect the broad definition of headache over a 12-month recall period and therefore does not necessarily indicate a high burden of clinically significant or recurrent headache disorders. Nevertheless, the finding suggests that headache symptoms are common among children in this setting and supports the value of further population-based research using standardized pediatric headache assessment.

The proportion of children classified as having migraine with aura also warrants attention. The relatively high proportion observed in this sample may be influenced by the small number of children meeting migraine criteria, and the characteristics of the original diagnostic instrument. In particular, the questionnaire was based on ICHD-II criteria and was validated in a tertiary headache population rather than in a representative community sample of children. Classification of aura symptoms in younger children may also be challenging because symptoms such as visual or sensory disturbances can be difficult for children or parents to characterize retrospectively. Therefore, the observed frequency of migraine with aura should be interpreted cautiously and should not be assumed to represent the distribution of migraine phenotypes in the general pediatric population. Future studies using contemporary diagnostic criteria and clinical assessment would be useful to determine whether this finding is reproducible.

A strong association was observed between reported family history of migraine and questionnaire-defined migraine. Children with a reported family history had approximately eightfold higher odds of meeting the questionnaire criteria for migraine than children without a reported family history. This finding is consistent with previous studies reporting familial aggregation of migraine [16,20]. However, the magnitude of this association should be interpreted cautiously. This finding supports an association between family history and questionnaire-defined migraine in this sample, but does not establish causality or quantify the independent effect of family history. Larger studies with multivariable analyses would be required to determine whether the association persists after adjustment for relevant demographic and clinical factors.

No statistically significant association was observed between migraine classification and sex. This finding should be interpreted cautiously because the study had a markedly uneven distribution of males and females and a relatively small number of children meeting the migraine criteria. Consequently, the absence of statistical significance does not necessarily indicate the absence of a true sex-related difference. The limited number of migraine cases also reduced the statistical power available for subgroup comparisons. Larger studies with more balanced recruitment would provide a more reliable assessment of sex differences in pediatric migraine in this population.

Several limitations affect the interpretation of the findings. First, participants were recruited using convenience sampling through an online questionnaire. This approach may preferentially recruit families who have greater interest in headache and families with specific medical knowledge. Consequently, the observed 14.8% proportion of questionnaire-defined migraine should be regarded as an estimate within the study sample rather than as a population prevalence estimate for children aged 5-12 years in Jeddah. The findings therefore have limited generalizability to children in the same age group.

Second, the Arabic version of the questionnaire was pilot tested for clarity but was not subjected to formal psychometric validation. Although pilot testing may identify problems with wording and comprehension, it does not establish whether the translated instrument retains the reliability, validity, sensitivity, and specificity of the original questionnaire. This limitation is particularly relevant because migraine classification was based entirely on questionnaire responses. Misclassification of migraine status, including the classification of aura, is therefore possible.

Third, the original questionnaire was based on ICHD-II criteria [18] and was validated in a tertiary headache population. Diagnostic criteria and clinical approaches to pediatric migraine have subsequently evolved, and a questionnaire developed and validated in a specialist clinical population may not perform identically in a community-based pediatric population. Thus, meeting the questionnaire criteria should not be considered equivalent to receiving a clinical diagnosis of migraine under current diagnostic standards.

Fourth, headache history was reported by parents or guardians over a 12-month recall period. This may introduce recall bias, particularly for less severe or remote headache episodes, and parents may not have been able to accurately characterize symptoms such as duration, associated features, or aura. The reliance on parent report may therefore have influenced both the estimated frequency of headache and the classification of migraine.

Fifth, migraine classification was not independently confirmed through clinical assessment by a pediatric neurologist or headache specialist. This limits the ability to determine the clinical validity of questionnaire-defined migraine and prevents assessment of alternative diagnoses or comorbid headache disorders. The relatively small number of children classified as having migraine also limits the precision of subgroup analyses, including analyses by sex and migraine phenotype.

Finally, the cross-sectional design prevents determination of temporal or causal relationships. In particular, the observed association between family history and questionnaire-defined migraine should not be interpreted as evidence that family history causes migraine.

Future epidemiological studies in Saudi children should prioritize representative school- or population-based sampling, larger sample sizes, and balanced recruitment across demographic subgroups. Use of contemporary standardized diagnostic criteria, preferably supported by clinical assessment, would improve the validity of migraine classification. Formal translation and psychometric validation of Arabic-language instruments should also be undertaken before their use in prevalence studies. Longitudinal or repeated-assessment designs could further reduce reliance on long-term recall and clarify the persistence and clinical course of pediatric headache. Such approaches would help determine whether the relatively high frequency of headache and the observed distribution of migraine phenotypes in the present sample are reproducible in the wider pediatric population of Jeddah and Saudi Arabia.

## Conclusions

In this study of children aged 5-12 years in Jeddah, 27 of 183 participants met the predefined questionnaire criteria for migraine, corresponding to a 12-month questionnaire-defined migraine prevalence of 14.8% (95% CI: 10.0-20.7%). These findings should be interpreted cautiously given the convenience sampling, reliance on parental questionnaire responses, and use of an Arabic-translated questionnaire based on ICHD-II criteria rather than contemporary clinical assessment. Further population-based studies using formally validated Arabic instruments and standardized diagnostic assessments based on current diagnostic criteria are warranted to obtain more representative estimates of migraine prevalence among children in Jeddah and Saudi Arabia.
